# Bringing the Community to the Uni—Critical Reflections on Youth Recreation Partnerships in Toronto

**DOI:** 10.3389/fspor.2022.876468

**Published:** 2022-05-17

**Authors:** Adam Ehsan Ali, Simon C. Darnell, Danielle Dinunzio

**Affiliations:** ^1^Faculty of Kinesiology and Physical Education, University of Toronto, Toronto, ON, Canada; ^2^Hart House, University of Toronto, Toronto, ON, Canada

**Keywords:** community, university, sport, recreation, partnership

## Abstract

The focus of this paper is an evaluation of a recreation project partnership between a co-curricular university department and various youth community programs in downtown Toronto, Canada. The goal of the Hart House Youth Community Recreation Project (YCRP) is to build a bridge between the university and its neighboring communities through recreation, arts, and dialogue-based programming that responds to the needs and interests of community partners and their youth members. Informed by the understanding that university/community partnerships are often paradoxical, the study assessed understandings of the program from the perspectives of the stakeholders involved. To do so, semi-structured interviews were conducted with the following two groups: organizers and leaders from the youth community programs, and staff members coordinating the project from the co-curricular university department. The results indicate that meaningful opportunities can be created within such partnerships through the provision of access to unique resources and recreation spaces; inclusion of partners in planning and program delivery; and through forging meaningful relationships between university staff and the program participants. However, significant challenges to creating and sustaining such opportunities also exist, including structural and social inequities that result in participants feeling othered in program settings; the instability and “delicacy” of trust within partnerships; and funding structure and resources. The findings shed light on, and make recommendations about, the potential benefits that youth organizations might gain from participating in university-community recreation partnerships, as well as the paradoxical nature of delivering and maintaining these partnerships.

## Introduction

This article presents an analysis of an ongoing partnership between recreation service providers at the University of Toronto (U of T), and community recreation organizations based in the city of Toronto. The paper is derived from a larger research project designed to assess, empirically and critically, the relationship between sport and social development for youth in urban Toronto, using participatory research methods. The project was funded in part by the Province of Ontario, Hart House, the center of co-curricular activity on the U of T campus, and through a mixture of other grants, and is aligned with a public policy mandate to leverage the social and economic benefits of sport.

This paper specifically analyzes the possibilities and limitations of organizing university/community partnerships to create sport-based social development opportunities for youth. To this end, we analyzed partnerships between five different community organizations and Hart House, enacted through the Hart House Youth Community Recreation Project (hereafter HHYCRP), which was started in 2016 as a bridge between the university and its neighboring communities through recreation, arts, and dialogue-based programming to respond to the needs and interests of community partners and their youth members. The five organizations captured in this paper all took part in the HHYCRP; they were: a public community Center, a Boys and Girls Club, a settlement services agency, an Indigenous support center, and a community center serving and supporting the LGBTQ2S community.

In what follows, we draw on data gleaned from interviews with the officials and leaders of the community organizations, as well as Hart House Staff who worked with these groups to facilitate the partnerships. Overall, the findings suggest that recreation partnerships between educational institutions like U of T and community organizations that support underserved youth can make important contributions to social development by increasing a sense of inclusion for community organizations and their participants, and supporting them to “take up new spaces” at institutions like universities that have historically excluded their communities. At the same time, such partnerships are often tenuous, and rest on an uneasy sense of trust—both inter-personal and inter-organizational—between stakeholders. Thus, university/community partnerships offer important institutional structures through which to facilitate sport-based social development for youth, but also require ongoing diligence and vigilance on the part of all involved. In this way, the results align with Strier (2014) call to embrace the paradoxical nature of university/community partnerships, the implications of which we discuss below.

The remainder of the paper is organized into six parts. In the next section, we offer a brief review of previous research on the topic of university/community partnerships, particularly those that are health and/or recreation-based, as well some key themes in sport-for-development that informed the research. This is followed by our theoretical framework, a description of the research context and the methods employed for the study. We then present the results, before a discussion of the implications. The conclusion suggests areas for future research.

## Literature Review

Since at least the 1990s, there has been a growing sense that partnerships between universities and community organizations can and should be created, extended and leveraged in order to improve research (see Begun et al., 2010; Ocean et al., 2021), facilitate more effective knowledge translation (see Hart and Wolff, 2006; Ginis, 2012), and better meet the needs of community members, especially youth (see Ostrom et al., 1995; Denner et al., 1999; Ardoin et al., 2014). As a term, university-community partnerships encompass numerous and diverse types of collaborative projects between a university and an external community, which can be broadly defined, represented, and operationalized; communities, for example, might be represented by neighborhoods, institutions, or other social groups (Strier, 2014, p. 156). Such partnerships have been documented between universities and the public education sector (Guerrero et al., 2013), not for profits (Minkler et al., 2008; Soleimanpour et al., 2008), health-related fields (Schroepfer et al., 2010), and members of marginalized or vulnerable communities (Suarez-Balcazar et al., 2004; Garcia et al., 2014; Anucha et al., 2020).

In large part, the need and desire for university/community partnerships has been driven by upheaval in the broader social safety net and a reduction in public funding, which has affected both public universities (like U of T) and community-facing organizations. As Strier (2014, p. 156) writes:

“*The development of the University Community Partnerships arena in the last decades has responded to huge social, economic and institutional transformations that have affected the academic and organizational cultures of research higher education institutions.”*

From this perspective, as public funding has become less reliable, and as forms of social capital that traditionally linked and stabilized educational and community organizations have waned, university/community partnerships have come to offer an innovative and flexible means for universities to achieve and broaden their teaching and research mandates, and for community partners to acquire the knowledge and expertise to improve their service delivery. While there are clear benefits to such strategies, they have taken place within the growing neoliberalization of higher education that has seen academia become increasingly commodified and market-focused, and has influenced the ways in which university partners have approached community engagement (Brackmann, 2015).

As Brackmann (2015) writes, it is important to consider how neoliberal logics shape the design and impact of university-community partnerships. Neoliberal scarcity creates the necessity of partnerships for community organizations who face limited operating funds, and the reasons that they turn to universities specifically as potential partners. As a result, such partnerships occur within unequal relations of power between the community and the university, which can undermine the effectiveness and intended direction of the partnership.

Neo-liberal logic can infiltrate the university side of such partnerships as well. Universities facing budget cuts are also often looking for partnerships that are either revenue-generating, or at least not overly expenses (Brackmann, 2015). Under such conditions, community organizations may find that looking to universities to supplement their resources or funding comes with transactional expectations. In fact, Hickey (2015) argues that amidst the evolution of the university into a corporatized entity, one that is often more interested in rankings and metrics of success and less focused on outcomes for the public good, community engagement has emerged as a strategy to help universities appear both interested and invested in the public without requiring serious reform. Community engagement has also provided new opportunities for academics to increase their academic success and visibility (Mtawa and Wangenge-Ouma, 2022). While compatible with neo-liberal ideals, these twin foci of corporate social responsibility for universities and individual aspirations for academics can undermine the transformative and justice-related aims of community engagement, reducing the likelihood that community members will see significant benefits (Mtawa and Wangenge-Ouma, 2022). Given this, in this study we were interested to see what benefits, if any, community members had accrued from the HHYCRP.

It is against this socio-political backdrop that university/community partnerships have become more recognized and common. The nature and focus of such partnerships can vary, but they tend to be built around the core competencies of post-secondary institutions, namely teaching, research/scholarship, and service (see Cook and Nation, 2016). Accompanying this increase in the practice of university/community partnerships have been analyses of their impact and efficacy. The results of these studies generally suggest that university/community partnerships can be effective for delivering and implementing evidence-based policies to communities (Spoth et al., 2013), strengthening the quality and experience of university-based researchers (Begun et al., 2010), and helping universities to establish their legitimacy and reputation within the surrounding community (Brusseau et al., 2015). The trend toward university/community partnerships has also extended to the field of health and physical activity (broadly defined), and researchers have similarly noted that such partnerships can be good for improving the overall quality of research outcomes, conducting more application-based research, and implementing effective interventions for behavior change. For example, such partnerships have been found to lead to lower substance misuse (Spoth et al., 2013), to promote new forms of physical activity within communities (Davis et al., 2017), support violence-prevention programs (Baker et al., 1999) and promote knowledge translation around physical activity for persons with a disability (Ginis et al., 2012). Overall, then, there is significant evidence to suggest that university/community partnerships can be beneficial to multiple stakeholders.

In turn, and whether focused on partnerships in general or the specific field of health and physical activity, most research into university/community partnership highlights similar basic principles that should be followed to maximize their benefits. These include: involving voices of multiple stakeholders; engaging in clear and collaborative planning; embracing patience; privileging community needs over academic outputs where applicable; considering multi-disciplinary approaches; conducting ongoing evaluations; and creating plans for sustainability and accountability (see Baker et al., 1999; Anyon and Fernández, 2008; Ginis, 2012; Brusseau et al., 2015; Davis et al., 2017, among others).

At the same time, important limitations of university/community partnerships have been identified. Foremost among these is that while university/community partnerships can make important differences in service provision and the experiences of various stakeholders, they are insufficient (at least on their own) for achieving or securing deep structural change at either social or political levels (Roussos and Fawcett, 2000). Thus, there is a tension within university/community partnerships themselves between practical and emancipatory approaches (or pragmatism vs. critical theory) (see Nation et al., 2011).

Notably for this study, the rise in popularity of university/community partnerships maps closely onto the growing popularity and overall institutionalization of sport in the service of social development. In this model of sport-for-development (SfD), highlighted by the global Sport for Development and Peace (SDP) sector (see Collison et al., 2019), sport programs are organized and implemented in order to meet non-sport goals like health promotion, gender empowerment and peace building or conflict resolution. While the general idea of sport making a positive contribution to social development has a long history, dating back as far as the 19th century (see Darnell et al., 2019), SfD has grown, both as a popular idea and as a policy model, in the past few decades. Importantly, and in a manner similar to university/community partnerships overall, the novelty of sport-for-development is due, at least in part, to the retraction of the welfare state across much of the global North, which charged educators and community leaders with the task of finding innovative ways to meet the needs of their students or community members (see Hartmann and Kwauk, 2011). Further, tensions similar to those in university/community partnerships exist in SfD, specifically between what has been termed the dominant model, which uses sport to teach participants how to survive amidst fundamental inequality, vs. the transformative model, which is less common and would mobilize critical pedagogy through sport as a way to agitate for broader and deeper social reform (see Hartmann and Kwauk, 2011). Thus, in this study we were interested in the extent to which the HHYCRP could be seen to move SfD from the dominant approach to the transformative.

## Theoretical Framework: The Paradox of University-Community Partnerships

In this paper, we embraced Strier (2014) contention that the partner-based relationships between universities and community partners are often paradoxical, and rarely either completely cooperative or conflictual, or reproductive or transformative. Instead, as Strier (2014, p. 157), writes university/community partnerships are paradoxical because while they are constructed to enable collaborative opportunities, it is also often the case that:

“*…each of the partners is motivated to promote and emphasize its own interests, at times at the expense of the others. The inherent complexity of partnership organizations lies in the fact that they are intentionally designed to face complex tasks by managing, integrating, balancing competing interests and demands in multifaceted institutional, economic and social environments.”*

As a result, Strier (2014, p. 162) advocates for viewing university/community partnerships through the theoretical conception of organizational paradox, which views “collaboration and conflict as formative and generative components” of university/community partnerships.

According to Strier (2014), there are seven areas of paradox in university/community partnerships, the first of which is the maintenance of “top-down” institutional legitimacy while ensuring community empowerment and autonomy in the partnership. The second area of paradox is the importance of relationship building, particularly across differences between university and community, against the outcome-oriented goals of the university. Third, the examination of power relations may lead to questions surrounding trust between the partners, which itself can lead to conflict or, if left unexamined, strengthen inequities in the partnership. A fourth and related paradox is the desire for an egalitarian approach, which might help foster stronger trust between partners, while at the same time adhering to the institutional hierarchies of the university. The tension between utilizing partnerships for transformational, long-term goals while maintaining tangible, short-term objectives, and aspiring to form a shared identity in the partnership and at the same time recognizing and responding to the different identities and experiences of community members make up the fifth and sixth paradoxes. The final paradox relates to the long-term sustainability of university-community partnerships, which relies on many factors, including changing political conditions, research agendas, funding opportunities, and student turnover (Arches and Apontes-Pares, 2005). The paradox here is that while the risk of turnover might threaten the continuity of the partnership, it could also provide the opportunity for vivacious energy brought on by fresh newcomers (Strier, 2014). Our analysis below embraced and applied this theoretical perspective to the HHYCRP.

In sum, this growing literature on the topic of university/community partnerships informed much of our analysis. That said, an important distinction is worth making; whereas most university/community partnerships are built around research, teaching or university-led service in the community, the focus of this paper, and the experiences captured below were notably different. What this project focused on was a university facility (in this case Hart House at U of T) engaging in partnership with community organizations in order to position itself as the site (both organizationally and physically) for youth to engage in programs and practices in support of their social development. In this sense, the specificities of the university/community partnership were crucial to this research, and we provide further details about this next.

## Research Context—Hart House Youth Community Recreation Partnership

As stated, the overall aim of the HHYCRP was to build a bridge between the university and its neighboring communities through recreation, arts, and dialogue-based programming that responds to the needs and interests of community partners and their youth members. This was done by facilitating community members' use of Hart House, a student-centered co-curricular facility that provides recreation, physical activity, and arts-based activities to U of T students, as well as adult-aged community members through paid memberships. The facility contains a number of amenities, including a fitness gym, indoor running track, basketball court, and swimming pool, and also functions as a revenue-generating space that hosts conferences, weddings, and other events.

Hart House itself holds a unique position at the University as well as within the city of Toronto. Built in 1919, the building is known for its dramatic Gothic architecture and its valuable art collection. It is often used as the backdrop for film and television and regularly features prominently in the university's promotional materials. Given its history and dramatic and intimidating look, and despite its literal accessibility to the university and community, Hart House does not hold a reputation for being open and friendly, especially for the diverse populations that comprise the downtown neighborhoods that surround the U of T campus.

It was specifically against this backdrop that the Hart House Youth Community Recreation Project (HHYCRP) began in 2016. The first iteration of the project was a partnership with the City of Toronto's forestry and recreation department, which grew eventually to include different community organizations. The project aligned with Hart House's overall mandate to increase its overall programming for underserved groups in three ways: by promoting inclusivity through helping young people to see themselves in university spaces; enhancing personal development through health, nutrition, and adventure education; and supporting social justice by aiding youth leaders in addressing the needs of their community.

The HHYCRP itself was operationalized in the following manner. The lead Hart House staff member for the HHYCRP met with staff-leads for each community partner to map out and decide upon specific programming goals and activities unique to that partner that would be delivered at Hart House. After that, formal meetings between the lead Hart House staff member for the HHYCRP and staff-leads for each community partner occurred on a regular basis—at a minimum of 3 times a year, but often more frequently—including a mid-term check-in and a debrief to review successes and areas for improvement. These meetings were further supported by informal communication in the form of emails, phone calls and in person conversations in order to maintain connection and rapport. As well, the HHYCRP hosted bi-annual meetings with Hart House staff and community organization staff to solicit feedback for integration into future programming choices. For most partners, the first or second program session would also include a youth brainstorming session, to gather youth participant input for integration into future programming decisions.

Operationalization also aimed to follow principles of flexibility and transparency. In the former, programs were designed with intentional gaps, or “flex days,” to allow for extra meetings with youth participants, to cancel or rearrange sessions, or respond to emerging issues, topics, or activities for which youth expressed interest. For the latter, facilitators or instructors hired to lead program sessions would be vetted by the lead Hart House staff member to ensure an appropriate fit for the youth partner group, and in some cases facilitators or instructors were asked to meet with community partners to ensure an appropriate fit before being hired to lead programs.

It is also worth noting that a number of practical and policy changes at Hart House were required in order for the HHYCRP to be implemented. These included: allowing persons under 18-year old to use the gym spaces, a shift that required communication with staff as well as Hart House members; new registration and sign-up policies and processes aimed at youth, and based on parental consent; the creation and posting of inclusive signage for bathrooms and changerooms, specifically at the request of the LGBTQ2S community center partner; training sessions led by community partners and aimed at Hart House staff so they could better understand partner needs; and regular presentations at Hart House Leadership Meetings and Hart House Board of Stewards to grow awareness and understanding of the HHYCRP.

Within this context, and given the literature discussed above, we were interested in the following research questions: What are the successes and limitations of partnerships like the HHYCRP for providing community recreation opportunities, particularly to traditionally underserved groups? What are the experiences, positive and/or negative, for community partner organizations who participate in the HHYCRP? And what implications or insights for future partnerships can be gleaned from the HHYCRP? To pursue these questions, we conducted semi-structured interviews, as discussed next.

## Research Methods

Ethical approval for this study was granted by the University of Toronto Research Ethics Board. Recruitment of interviewees began with an announcement during a regularly scheduled HHYCRP meeting attended by community leaders, Hart House staff, and members of the research team. In recruiting interview participants, we explained our interest in learning more about the partnership between Hart House and the community organizations in order to evaluate the impact, successes, and challenges of the HHYCRP.

While participating in research was not a requirement of community partners, some level of rapport between the researchers, Hart House staff, and community partner leaders had been previously established, through the HHYCRP itself as well as prior participatory research activity (see Smith et al., 2021), which enabled successful recruitment. Following the initial announcement, follow-up recruitment, with both community leaders and Hart House staff, was conducted through email, and interview dates and times were scheduled at a mutually convenient place and time.

The initial interviews were conducted in person, but the COVID-19 pandemic occurred in the middle of the recruitment/interview process, and as such subsequent interviews were scheduled and completed on Zoom. Before each interview commenced, participants read the information letter, and gave written consent to be interviewed and recorded. In total, 14 participants were recruited for this study: seven community group leaders from four different partner organizations and seven Hart House staff members who were involved in the project. The Hart House staff interviewed ranged from front-line staff to those at the managerial level.

Semi-structured interviews were conducted individually with the community partner (CP) leaders and Hart House staff (HS), with each interview lasting between 50–90 min. After the interviews were transcribed verbatim, they were imported into NVivo for analysis. Analysis began with an initial coding process, whereby we identified initial major themes. In this stage, we used open, axial and selective coding, as described by Corbin and Strauss (1990). We also derived a coding framework in the initial interviews that helped to inform the analysis of subsequent interviews, while remaining attentive to new themes that developed later on in the research process. As such, our process was iterative and mobilized both inductive and deductive forms of interpretation. Based on this process, similar themes were grouped together and we put the coded data into conversation with the existing literature on university-community partnerships for further analysis. We drew connections between the relevant scholarship and the themes that emerged from our initial coding process. In the next section, we present the main findings, grouped into three major themes: Inclusionary Partnerships, Taking Up Space, and Structural Inequalities.

## Results

The findings of the interviews revealed that the HHYCRP helped create important opportunities for participation in sport and non-sport related recreational activities in post-secondary spaces, and also led to important changes at the University itself. At the same time, significant barriers, both material and non, continued to preclude meaningful inclusion of program participants within the university community. Here we discuss three main themes that describe this overall finding: the inclusionary nature of the institution-partner relationship that resulted in meaningful opportunities and access to programming for the beneficiaries; the importance of “taking up space” within university environments; and ongoing structural and racial inequities resulting in a sense of precarity within the overall partnership. Subthemes are also listed within each main theme.

### Inclusionary Partnerships: Goals, Access and Opportunity

A clear goal of the program, and one which was consistently heralded by the community partners, was to champion a participatory approach to the HHYCRP that helped shape, improve, and enhance program delivery. This approach helped increase opportunities for participants to engage in activities and spaces that they otherwise might be unable to access, such as sport gymnasiums, arts-based activities, and educational workshops. The community partners collectively stated that having an institution provide free access to sports gyms was a valuable asset to their program offerings. For example, CP1 explained that it can be difficult to find external spaces that are affordable for their non-profit organization within a limited budget, but that through this partnership they were able to offer sport-based programming. Similarly, CP2 stated that “it was important that we offer them new opportunities… and also, opportunities that maybe could lead them to other ways of thinking.” Arts-based and education-based programming were also considered integral to creating opportunities for the beneficiaries to think differently about the world, themselves, and what they might be interested in pursuing in relation to education or potential careers.

Another goal of the partnership was to provide opportunities for the participants to spend time on a university campus, which was seen as particularly important given that the youth embodied a mix of demographics that have typically been excluded from post-secondary spaces. The goal was to support participants' comfort levels within the university so that they might see themselves as legitimately belonging within such spaces. By spending more time in the institution participating in sport and arts-based programming, the hope was that post-secondary education would eventually feel less overwhelming and more achievable in the eyes of the youth participants. CP4 explained that “with the partnership… it was really to break down those negative stereotypes that the participants might have felt about being a part of post-secondary education…,” while HS2 stated that “what this program can do is provide opportunity in terms… if graduates of the program to any post-secondary and they're (the) first in the family (to do so), it's a win.”

#### Programming

A major factor that helped to lead to these opportunities was the inclusionary partnership approach, as leaders of both the institution and the community partners worked together to create meaningful programming that served the needs and desires of the beneficiaries based on their experiences and backgrounds. Community partner leaders noted that programming was at its best when they, as partners, were included right from the beginning of the planning process, starting with the preparation and planning, the execution of the program, and in the evaluation and follow-up from the institution. CP3 stated that this collaborative approach and process was highly valued; in particular they were asked for feedback on the feasibility of certain programming “based on past experiences (of) what would be most important for the youth.” CP1 further explained that the institution partners would check in at least twice a month, and that they were “very accommodating to any request I have… and the youth (beneficiaries)… see that.” These sentiments were echoed by the institution staff who saw community partners as equal contributors to the program planning. HS1 explained that “the model of how we work with our community partners is… driven by their requests and to try to be really responsive to what the youth who are in those programs are asking for in terms of… opportunities.”

Importantly, to maximize the benefits to the youth, an inclusionary approach was also required by the instructors who delivered the programming. CP3 explained that programming could be transformational when the instructors included learning about the beneficiaries and the organization as part of their preparation. In one example, they described how an instructor leading a meditation workshop organized it through an Indigenous lens, which reflected the experiences of the youth. She discussed the needs of the beneficiaries with the leaders beforehand, memorized a land acknowledgment, and prepared a smudge ceremony as part of the workshop delivery. CP3 stated that this type of preparation was “incredibly important… for the youth when they see someone who's never met them before… who's invested so much into working with them… it increases their self-esteem and makes them feel like they're valuable.”

#### Routine and Structure

Also important for the inclusionary partnership was establishing routine and structure early in the planning process as well as the execution of the programming. Interviewees stated that this led to higher levels of comfortability and feelings of belonging amongst the participants. The community partners also indicated that participation levels tended to be much higher when the youth knew what the activity was going to be each week during their time in the program, the day and time it was scheduled for, and how often the programming was scheduled for more generally. Of particular note was that both institution staff and community partners' perceptions of the second year of the partnership were more positive, which they attributed to the more structured, formalized approach to the program as compared to the previous year. The community partners noted that “structure is very important even though it's a drop-in program” CP1, and that “one of the reasons the program… is successful is because they know exactly what they are doing every day and they need that… it took away that apprehension factor, I think” (CP3).

#### Staff Interactions

In addition to the importance of the inclusionary planning process that was discussed by the interviewees, the community partners overwhelmingly endorsed the ways in which the institution's front-line staff, such as those who worked at Hart House's welcome desk, interacted with them and the youth participants. The partners stated that, in addition to the flexibility and adaptability of the administrative staff that led to the success of the planning process, the front-line staff were very positive, supportive and receptive in assisting the youth and program leaders during their program time. This was integral to helping the youth feel comfortable participating in the activities. CP2 explained that the student interns who had done the workshops with the students had been really receptive and energetic, and that “our youth sense that and I think that's why they gravitate to them and ask them questions as we're doing the activity.” CP1 also stated that the staff are “not looking at us like ‘what are you doing here’… they're always open if I have any questions or need to know where a room is, they've been very respectful.”

### Taking Up Space and Visibility

The second major theme from the results was the importance of the youth taking up space in Hart House. Both the institution's program staff and community partner leaders also discussed the significance of the participants being meaningfully involved in activities within the physical activity and arts-based programming such that they might be regarded as visibly active members of the university community. This was considered an integral outcome of the partnership because, at least symbolically, it challenged traditional ideas of who belongs within university spaces. One of the partner leaders suggested that there “is a very visible lack of racialized bodies, queer bodies, poor bodies in that space so I think part of it was this hope … (to) have people be like no, take up space here, this is for you… which I don't know exists… in the absence of these types of initiatives” (CP5).

Visibility was also important because it helped the overall participant demographics at the institution better reflect the communities that surround the university but have been historically excluded from it or made to feel unwelcome. In this sense, the partnership was important for challenging dominant representations of belonging, because the university “has these great facilities that… (but) a lot of marginalized folks will never see or have access to (them) when they live just like a block or two from these spaces” (CP5). The partnership was therefore perceived as one strategy for overcoming such barriers by integrating community members within university programming. In describing a large group activity that their youth took part in, CP6 explained that “there's something really powerful about us… occupying the space…,” particularly for the ways that it symbolized changing representations of community-based users.

#### Feelings of Belonging and Broadening Mindsets

Moreover, by occupying space on a more regular basis in the institution, interviewees felt that the youth would come to see themselves as meaningful members of the post-secondary environment. The Hart House staff also sought to create a welcoming atmosphere to help address this objective. HS3 explained that the staff should “do whatever we can… to ensure that people know that they are welcome and that they are seen… that their experiences, their identities are understood and reflected back at them in our physical spaces, in our programs.” HS1 similarly added that staff hoped that participants would feel more welcome on campus, and see themselves reflected in those spaces more often, through the partnerships.

These perspectives aligned with the results of the interviews with community partners. One of the community leaders explained that a goal was to “create a familiar setting in an unfamiliar place” (CP7) by exposing their participants to the opportunities that post-secondary spaces have to offer, and they saw the partnership as important in achieving this. These feelings of unfamiliarity were captured when participants from CP2's organization first took part in the programming at the institution; they stated that “you saw the reactions on their faces like ‘well I don't belong there, I don't see myself there’.” CP2 stated that one of the most important parts of the program was therefore to respond to this discomfort or inadequacy in order to help forge belief in their youth that they indeed belonged in these spaces. Through the ongoing partnership, CP2 suggested that a sense of comfort and belonging had grown. And CP3 stated that they saw this as the primary goal of the partnership, to “strive to have impact on them (the youth) and to provide those opportunities and to open doors… and help them to… feel like they're valued and that they're important….”.

In turn, the increased visibility of the participants in the spaces of the institution was not only important for better reflecting the surrounding communities of the university, but also to broaden the mindset of those deemed to be the “traditional” members of the institution (including both university students and community members who tend to be older, white, and male). One of the institution staff explained that when considering the older, senior members of the physical activity space in the facility, “sometimes the default in that space feels like okay, this is a serious place” (HS1). Conversely, having beneficiaries in the space who were younger, racially diverse and from different class backgrounds served to introduce a different energy that HS1 believed is “really healthy” for the institution.

It is also important to note that in addition to the positive experiences of community partners captured here, practical changes were made at Hart House as a result of the HHYCRP. These included: block scheduling holds placed on gym spaces dedicated to youth/community access; working with an Indigenous Artist to re-design spaces for youth programming; and creation of a cross-departmental Access Implementation Team to support short and long-term planning for the HHYCRP. All of these changes illustrate recognition of the success of the HHYCRP to date, and commitment to continue enabling such work into the future.

### Ongoing Structural Inequities

Despite the successes of the program noted above, there was still a sense amongst the interviewees of ongoing challenges, particularly concerning structural inequities in the program (both historical and contemporary) that impacted participants' experiences. Many community partners noted that, at times, the youth participants felt intimidated and out-of-place, particularly during the initial stages of the partnership. While we noted the positive feelings of belonging earlier, it is important to acknowledge that these feelings were not static, but rather dynamic and malleable based on particular factors within the institution. These included the physical infrastructure of the institution, which lacked accessible entryways, its gothic architecture exterior and colonial interior that respondents noted as being unwelcoming to non-white communities, and locked doors to empty rooms that gave off a cold impression to those wishing to access “hangout” spaces. HS4 noted that the built architecture implicitly welcomed a certain kind of Hart House member, which was unlikely to be embodied by the youth. They explained one instance when they were leading the community partner group to a part of the facility for their activity, one of the participants “shushed his friend for being too loud.” HS4 explains “it's not like there was a conference taking place like it, so it's just like we, the buildings, the campus like creates this weird like everybody is watching you, be on your best behavior.”

#### Membership Prestige and Race

Additionally, while front-line staff were described as very welcoming by the community partners, respondents also noted instances where Hart House members and university students made comments toward or about the participants that carried racist, classist, and ageist undertones. These included instances of conflict between the community youth participants and Hart House members over the use of basketball courts, as well as indirectly through conversations with Hart House staff. In the latter, Hart House members were heard asking who the youth were, why *they* were present, and whether *they* were in fact allowed to book time? On another occasion, a staff member from another institution with the university sent an email to one of the HS reminding them about ratios and supervision and to ensure that their group, which were composed of young black teens, cleaned up after themselves. This marked the first communication of any such reminders, raising questions of its motivation.

Altogether these types of interactions illustrated a discourse of membership prestige, in which “traditional” members of the institution communicated in ways that demarcated themselves as entitled to institutional spaces, and in contrast to the program participants. These were complicated by the fact that Hart House's fitness center has an 18+ age membership policy and thus has not been a space frequented by children and youth. To address this issue, many of the staff respondents noted the importance of educating the current membership about the changes in programming. This included communicating the institution's commitment to the community, informing the membership about the program, and relaying requests for all members of the institution to adapt to such changes.

#### Invisible Gates of the University

At the same time, CP and HS respondents also noted the challenges that came from Hart House's association to the larger university, and the reputation the latter has within the surrounding communities in which many of the participants reside. CP2 explained, for example, that “I think (university) needs to find a way not to be known as… the old white establishment university,” and to ask itself “who feels like I deserve to apply (here).” CP5 noted that “it is an old white rich boys' club and that's painfully clear…. I appreciate the work that… many people are doing to… ensure that is not it's future its roots are real deep.”

These excerpts from the community partners were supported by the institution's staff, who acknowledged its colonial roots and the negative impact on newcomers wishing to access their services. HS3 stated that the institution has historically been an exclusive domain for “well-heeled, private school” males, acknowledging that women were restricted from its spaces up until the 1970s. The construction of the university as unwelcoming and potentially hostile to members of its surrounding communities created what interviewees described as an invisible gate or invisible bubble that surrounds the campus. Neighboring residents, rather than moving through the campus to access another part of the city, would instead go around its perimeter. HS1 explained that this would occur “because the kind of psychological barriers or the lack of familiarity of that space and feeling like it's not a space that's open to you even though we… might be centered in the middle of the downtown.” HS4 added that “the (invisible) walls to the university are quite high for people that live really close and who could… access the resources we have.”

#### Partnership-Precarity and Trust-Building

The challenges described above point to the importance of building and maintaining trust between the community organizations and the institution, particularly because there is a clear unequal power relationship between the two. The CPs pointed to the inclusive program planning as one important element of the trust-building that has benefitted the relationship, but at the same time expressed caution regarding the psychological safety of their participants within these spaces. For one community partner, this meant making sure that their participants would not be re-traumatized through their participation in the program, which they did by having difficult but thoughtful conversations early on with the institution staff about how to make the programming as inclusive as possible for the participants (CP5).

At the same time, however, some HS also expressed concern that this precarity may have resulted in the CPs likely withholding critiques of the program or institution in order to avoid straining the partnership, and potentially losing opportunities for their participants. HS4, for example, speculated that community partners likely felt that “I don't want to say anything because I don't want to jeopardize the relationship.” In this sense, a discourse of gratefulness existed, even in instances where institution staff solicited constructive criticism or information about ways to improve the program. One of the community partners, CP7, largely confirmed this when asked about the ways to improve the partnership, responding “they're giving us an opportunity that no one else has given us… and… we wanna bring really cool experiences to (them)… and showcase our gratitude to them…”.

This sense of precarity likely also extended to the youth participants, whose behavior expressed an understanding that their access to these spaces was also conditional. Institution staff tried to respond to these concerns by communicating to the youth that their participation was not dependent on demonstrating “good behavior” within the space as though they were being surveilled. Examples like that of one youth shushing his friend while in the space exemplified the work still to be done in order to build such a culture.

#### Financial Precarity and Staff

Finally, precarity was evident in that institution staff acknowledged the dependence of the partnership upon various forms of funding, which were not guaranteed. This included the non-permanent employment status of the lead HHYCRP staff member, the 5-year grant to support the evaluative research, as well as the precarious financial status of community partners. HS5 stated that “in order to… have these community programs… we need to bring in enough of the paying business… we have to have the resources to be able to support it… and… we only have so much space.” Moreover, there was agreement amongst the institution staff respondents that there currently exists limited personnel to respond to the growing demands of the program, and that the partnership might require further institutional support in order to gain long-term sustainability.

## Discussion

There are several connections between the results of our study and Strier (2014) areas of paradox in university community partnerships. First, as Strier (2014) states, a healthy and genuine partnership requires a bottom-up, grassroots approach, which means that “academic authorities must voluntarily give up a great deal of institutional control over the management of the partnership” (p. 158). This was observed in the lengths that Hart House administration went to collaborate with community partners on project programming, and its willingness to adjust its priorities based on the changing circumstances of the partner. At the same time, the revenue-generating nature of Hart House meant that the partners also had to adjust based on scheduling that could be made available while not affecting “business-as-usual.”

Second, the quality of relationship-building vs. the maintenance of organizational effectiveness paradox was illuminated by the HHYCRP when youth participants were confronted with traditional community members and university students in the Hart House space. While this interaction could represent a “an alignment of partners that never have acted in a shared manner had the partnership not been established” (Strier, 2014, p. 159), the hostility toward the youth demonstrated the need for clear communication and support from Hart House staff to manage such conflicts. This strategy was required not only to improve relationships with the community partners, but also to manage the expectations of Hart House members and university students who pay to use the space. At the same time, the results captured in this study would seem to suggest that the act of partnering can be positively transgressive, because it brings people to places in which they might not be, and affords them experiences they might not have otherwise. From this perspective, while partnerships like the HHYCRP can be useful for confirming the reputation of facilities like Hart House and institutions like the University of Toronto, they also challenge the insularity of these spaces in productive ways.

Third, the findings revealed a delicacy of trust between the community partners and Hart House, which was driven by an unequal relationship of power whereby the partners were dependent on the institution for access to space, programming, and resources they otherwise may not have. This tension speaks to the paradoxical nature of evaluating unequal power relations and building trust within partnerships (Strier, 2014). Community partners recognized their relatively subordinate position within the partnership hierarchy and felt some need to maintain this position to ensure that the opportunities afforded to them might continue. This, however, meant that the trust within the partnership was itself precarious. At some level, this paradox may have been unavoidable within the structure of this particular partnership. Interestingly, however, many HS felt such an examination of power relations was important, which aligns with Strier (2014) notion that such partnerships require efforts to explore these competing demands, while CPs generally chose not to explore this. This seems to align with Brackmann (2015) findings that community organizations did not express concern regarding these unequal power relations because the partnership provided solutions that met their immediate needs (p. 134).

This tension regarding the short and long-term goals of the HHYCRP also falls within Strier (2014) fields of paradox, which was realized most in the balance between meeting these immediate needs of the community organizations while aspiring to transformational outcomes. While Hart House served as an excellent opportunity for participants from community partners to engage in physical and arts-based activities on a semi-regular basis, and to help them see themselves as belonging in post-secondary spaces, it is less clear whether these more tangible achievements might result in larger institutional shifts within Hart House or the university more broadly. The paradox here also lies in the fact that while providing these opportunities to youth participants is meaningful, they do not address the structural inequities that shape the need for such provisions in the first place.

There are also clearly some immutable tensions of inclusion/exclusion within a space like Hart House as the site of community partnership. Indeed, the very spaces of Hart House that were made available to community partners through the HHYCRP were the products of colonial practices, and deeply embedded within ideologies of exclusion. While the “occupation” of these spaces through partnership can be an act of resistance, it can also serve as a reminder and acknowledgment of the structures of exclusion themselves. Following Strier (2014), we would concur that in the face of such tensions, the aim should NOT be to try and transcend the paradoxical, but rather to encourage, prepare and support all stakeholders in this kind of partnership to embrace the existence and potential benefits of paradox. As Strier (2014, p. 163) writes:

*In order to handle with high degrees of complexity which are intrinsic characteristics of partnerships, University Community Partnership's primary premise is therefore to establish a critical and reflective organizational culture in which participants advance a collective mindset centered on the theoretical and practical embrace of paradox*

The results of this study suggest the need to move toward, not away from, the paradoxical complexity of partnership.

In addition to the implications related to Strier's fields of paradox, there are other implications stemming from the analysis. First, and with respect to sport-for-development, the results suggest that university/community partnerships are important and necessary because organizing and implementing sport and recreation programs and policies to support the social development of youth is often beyond the scope of a single organization. In this case, neither Hart House nor the partner organizations had the means, connections, time or capacity to deliver complete programs to youth independently, but the HHYCRP provided a structure through which this could occur. Irrespective of the results of the program on participants, the utility of the partnership model in this case deserves to be recognized. This, in turn, suggests that there is room for universities like U of T, co-curricular centers like Hart House, and even faculties of Kinesiology or sport science to expand the scope of what they do and who they serve. There certainly seems to be an appetite and opportunity to deliver recreation opportunities to community participants through the structures, resources and facilities of the university, and, following Brusseau et al. (2015), this would seem to provide important opportunities for universities to establish legitimate reputations for supporting the health and well-being of the members of the communities in which they operate.

Indeed, there would be appear to be significant potential to extend the HHYCRP in notable ways. For example, as Anyon and Fernández (2008) suggest, university/community partnerships can include site-based placements of university staff, which in turn can lead to important, critically informed knowledge production for researchers and practitioners alike. As applied to the HHYCRP, this might mean that in addition to welcoming community partners onto the U of T campus, U of T would go into the community, working with and/or for the partner organizations to deliver sport-based social development programs for youth. In fact, and despite barriers due to the COVID-19 pandemic and staff capacity, Hart House currently delivers a limited version of this programming in recognition of the importance of meeting partners in their spaces to build strong trust and bonds. This kind of activity can be particularly achievable and successful if it utilizes the ‘linking capacity agents’ framework described by Spoth et al. (2004), in which intermediaries work to bridge the needs of universities and community organizations.

Finally, we suggest the importance of reflecting on one of the longstanding criticisms applied to the broader SfD context, and that is also relevant to the HHYCRP, namely that in the dominant model, participants are provided with survival skills while programming stops short of challenging the overall structures of inequality (Hartmann and Kwauk, 2011). From this perspective, it is worth reflecting on the extent to which partnerships like the HHYCRP are illustrative of the dominant, transactional model of SfD as opposed to a transformative one. University/community partnerships are beholden to such politics, not beyond them. As Roussos and Fawcett (2000) argue, in the mobilization of university/community partnerships to support health, there is still the need to investigate and even challenge the social determinants that lead to unequal health outcomes in the first place. Our research suggests a similar necessity in navigating the structures of youth recreation provision through university/community partnerships. In the context of the HHYCRP, our results show that the desire for a transformational partnership appears to be one-sided. That is, our interviews with HS partners demonstrate a strong motivation for long-term commitment and mutual immersion, albeit with funding and capacity to fulfill that commitment still pending, while the CPs indicated how the partnership was able to help them fulfill their own organizational goals. The latter, which best captures the partnership as it currently stands, aligns with Strier (2014) argument that transactional partnerships may be used to achieve individual partner's objectives through exchange, but that the beneficiaries may remain unchanged.

## Conclusion

In this paper, we have argued that partnerships like the HHYCRP can positively impact social development amongst underserved youth through the provision of opportunities to participate in sport, recreation, and arts-based activities within university settings. It can also help institutions like universities to transform in positive ways. This was realized through our project findings, which revealed that such partnerships can have success through inclusionary processes where community partners are deliberately and centrally involved in the planning, execution, and evaluation of the project, and when institution staff engage with participants in positive, supportive, and receptive ways. These processes were integral to creating accessible, meaningful, and structured programming for the participants, such that they could actively and more comfortably engage in activities within Hart House. This helped increase the capacity of the youth to take up a space that has historically excluded them, create a sense of belonging within the participants, and symbolically challenge dominant representations of the university community.

At the same time, community partners and youth participants had to navigate longstanding structural and racial inequities embedded within both the physical and cultural architecture of Hart House in ways that limited the project's outcomes. These inequities extended to the larger university, which was perceived as an elitist, colonial institution was welcoming to few, prestigious members and hostile to outsiders. The resulting tensions demonstrate the importance in building and maintaining trust between the university and community organizations, while acknowledging that unequal relationships of power exist between these partners; particularly in that Hart House is able to provide resources and space that would otherwise be inaccessible for many of the community organizations.

These results also demonstrate that university-community partnerships exemplify the necessity of an interorganizational approach to providing sport and recreation programming that supports the social development of youth, and that such partnerships can also help each organization more effectively meet their goals than if pursued in isolation. Moreover, there are clear opportunities to enhance the HHYCRP by mobilizing university staff, researchers, and students to support the work of community partners in their physical activity and recreation spaces. At the same time, continuing to welcome and support community partners and youth within the walls of Hart House remains a necessary and important initiative toward creating transformative cultural shifts within the university.

It is also important to acknowledge a significant limitation of this paper, which is that the voices of the youth participants were not included in this phase of the project. As such, the conclusion we draw regarding the participant's feelings of belonging within Hart House, for example, were derived from our interviews with community partner leaders. While these leaders work very closely with the participants throughout and well-beyond the partnership, they are still only communicating their perceptions of the youth's feelings toward Hart House. It should be noted that the participants have been included in earlier phases of the project (see Smith et al., 2021). A follow-up study involving interviews or focus groups directly with youth participants would help address this limitation and provide a fulsome understanding of the partnership, its benefits, and ongoing challenges. At the beginning of the study, we had also planned on conducting a survey with Hart House community members and students that would have provided more direct knowledge of their experiences of the HHYCRP. The COVID-19 pandemic, however, shut down the university before the survey could be finalized and released. Given that many of the challenges identified in our interviews involved these two social groups, hearing their perspectives would shed further light on prevailing understandings of who belongs within the university space. To this end, we believe that more research focusing on the racialized dynamics of university-community partnerships is required to further illuminate the ways such tensions play out in practice.

## Data Availability Statement

The raw data supporting the conclusions of this article will be made available by the authors, without undue reservation.

## Ethics Statement

The studies involving human participants were reviewed and approved by University of Toronto Research Ethics Board. The patients/participants provided their written informed consent to participate in this study.

## Author Contributions

All authors listed have made a substantial, direct, and intellectual contribution to the work and approved it for publication.

## Funding

This work was supported by Early Researcher Award, Province of Ontario, 2016–2021 $100,000 - Using Participatory Action Research to Understand Sport and Social Development in Toronto, Ontario ER15-11-063.

## Conflict of Interest

The authors declare that the research was conducted in the absence of any commercial or financial relationships that could be construed as a potential conflict of interest.

## Publisher's Note

All claims expressed in this article are solely those of the authors and do not necessarily represent those of their affiliated organizations, or those of the publisher, the editors and the reviewers. Any product that may be evaluated in this article, or claim that may be made by its manufacturer, is not guaranteed or endorsed by the publisher.
